# Incidence, clinical features and perinatal outcome in anomalous fetuses with late‐onset growth restriction: cohort study

**DOI:** 10.1002/uog.24961

**Published:** 2022-11-01

**Authors:** A. Dall'Asta, T. Stampalija, F. Mecacci, R. Ramirez Zegarra, S. Sorrentino, M. Minopoli, C. Ottaviani, I. Fantasia, M. Barbieri, F. Lisi, S. Simeone, R. Castellani, A. Fichera, G. Rizzo, F. Prefumo, T. Frusca, T. Ghi

**Affiliations:** ^1^ Department of Medicine and Surgery, Obstetrics and Gynecology Unit University of Parma Parma Italy; ^2^ Unit of Fetal Medicine and Prenatal Diagnosis Institute for Maternal and Child Health IRCCS Burlo Garofolo Trieste Italy; ^3^ Department of Medicine, Surgery and Health Sciences University of Trieste Trieste Italy; ^4^ Department of Biomedical, Experimental and Clinical Sciences, Division of Obstetrics and Gynecology University of Florence Florence Italy; ^5^ Department of Obstetrics and Gynecology, University Hospital Rechts der Isar Technical University of Munich Munich Germany; ^6^ Department of Clinical and Experimental Sciences, Section of Maternal and Child Health University of Brescia Brescia Italy; ^7^ Division of Maternal and Fetal Medicine University of Rome Tor Vergata Rome Italy

**Keywords:** aneuploidy, CGH‐array, congenital malformation, fetal growth restriction, perinatal outcome, respiratory complication

## Abstract

**Objective:**

To describe the incidence, clinical features and perinatal outcome of late‐onset fetal growth restriction (FGR) associated with genetic syndrome or aneuploidy, structural malformation or congenital infection.

**Methods:**

This was a retrospective multicenter cohort study of patients who attended one of four tertiary maternity hospitals in Italy. We included consecutive singleton pregnancies between 32 + 0 and 36 + 6 weeks' gestation with either fetal abdominal circumference (AC) or estimated fetal weight < 10^th^ percentile for gestational age or a reduction in AC of > 50 percentiles from the measurement at an ultrasound scan performed between 18 and 32 weeks. The study group consisted of pregnancies with late‐onset FGR and a genetic syndrome or aneuploidy, structural malformation or congenital infection (anomalous late‐onset FGR). The presence of congenital anomalies was ascertained postnatally in neonates with abnormal findings on antenatal investigation or detected after birth. The control group consisted of pregnancies with structurally and genetically normal fetuses with late‐onset FGR. Composite adverse perinatal outcome was defined as the presence of at least one of stillbirth, 5‐min Apgar score < 7, admission to the neonatal intensive care unit (NICU), need for respiratory support at birth, neonatal jaundice and neonatal hypoglycemia. The primary aims of the study were to assess the incidence and clinical features of anomalous late‐onset FGR, and to compare the perinatal outcome of such cases with that of fetuses with non‐anomalous late‐onset FGR.

**Results:**

Overall, 1246 pregnancies complicated by late‐onset FGR were included in the study, of which 120 (9.6%) were allocated to the anomalous late‐onset FGR group. Of these, 11 (9.2%) had a genetic syndrome or aneuploidy, 105 (87.5%) had an isolated structural malformation, and four (3.3%) had a congenital infection. The most frequent structural defects associated with late‐onset anomalous FGR were genitourinary malformations (28/105 (26.7%)) and limb malformation (21/105 (20.0%)). Compared with the non‐anomalous late‐onset FGR group, fetuses with anomalous late‐onset FGR had an increased incidence of composite adverse perinatal outcome (35.9% *vs* 58.3%; *P* < 0.01). Newborns with anomalous, compared to those with non‐anomalous, late‐onset FGR showed a higher frequency of need for respiratory support at birth (25.8% *vs* 9.0%; *P* < 0.01), intubation (10.0% *vs* 1.1%; *P* < 0.01), NICU admission (43.3% *vs* 22.6%; *P* < 0.01) and longer hospital stay (median, 24 days (range, 4–250 days) *vs* 11 days (range, 2–59 days); *P* < 0.01).

**Conclusions:**

Most pregnancies complicated by anomalous late‐onset FGR have structural malformations rather than genetic abnormality or infection. Fetuses with anomalous late‐onset FGR have an increased incidence of complications at birth and NICU admission and a longer hospital stay compared with fetuses with isolated late‐onset FGR. © 2022 The Authors. Ultrasound in Obstetrics & Gynecology published by John Wiley & Sons Ltd on behalf of International Society of Ultrasound in Obstetrics and Gynecology.

## Abstract 2

Linked article: There is a comment on this article by Li and Lei. Click here to view the Correspondence.

**CONTRIBUTION**

**What are the novel findings of this work?**

This research adds to the knowledge of the incidence, clinical features and perinatal outcome of fetuses with anomalous late‐onset fetal growth restriction (FGR). The majority of cases with anomalous late‐onset FGR are associated with isolated structural malformations. Anomalous late‐onset FGR is associated with a higher incidence of respiratory complications at birth and admission to the neonatal intensive care unit (NICU) and with a longer stay in the NICU compared with non‐anomalous late‐onset FGR.

**What are the clinical implications of this work?**

The identification of anomalies in the context of late‐onset FGR is associated with an increased incidence of composite adverse perinatal outcome, a 3‐fold higher incidence of need for respiratory support at birth and a 10‐fold higher incidence of need for intubation compared with cases with non‐anomalous late‐onset FGR. Such information is important for parental counseling in pregnancies complicated by anomalous late‐onset FGR.

## INTRODUCTION

Fetal growth restriction (FGR) is defined as the inability of a fetus to reach its biological growth potential^1^, ^2^. FGR is currently classified as early onset when diagnosed prior to 32 + 0 weeks' gestation or late onset if the diagnosis is made at or beyond that gestational age^3^, ^4^. Late‐onset FGR is estimated to account for approximately 80% of all cases of FGR^4^, ^5^, and is acknowledged to represent a risk factor for adverse perinatal outcomes^6^, ^7^ and long‐term health consequences, including cardiovascular morbidity and mortality and neurodevelopmental delay^8^, ^9^.

FGR is most commonly secondary to impaired placental function^10^, ^11^, however, it may also be associated with fetal malformations, either isolated or in the context of genetic syndromes, aneuploidies or congenital infections^12^, ^13^. Data regarding the etiology and perinatal outcome of FGR diagnosed at periviable gestational age (22 + 0 to 25 + 6 weeks) and associated with fetal anomalies or congenital infections have been reported before^14^, however, to our knowledge, there is little or no information on the incidence and prognosis of fetal smallness diagnosed after 32 weeks and associated with fetal anomalies. The aim of this study was to describe the incidence and clinical features of late‐onset FGR associated with genetic syndromes or aneuploidy, structural malformations or congenital infection (i.e. suspected anomalous late‐onset FGR) and to compare the perinatal outcome of these cases with that of non‐anomalous late‐onset FGR fetuses.

## METHODS

This was a retrospective, multicenter cohort study involving four academic tertiary maternity units in Italy (University Hospitals of Parma, Florence, Brescia and Trieste) and which included a consecutive series of fetuses diagnosed with late‐onset FGR between 2011 and 2021. All pregnancies were dated based on first‐trimester crown–rump length measurement.

For the purposes of the study, an electronic search of the ultrasound databases of the four maternity units was undertaken. All singleton pregnancies referred or followed‐up between 32 + 0 and 36 + 6 weeks' gestation owing to suspected fetal smallness were screened, aiming to identify those with late‐onset FGR, as defined by abdominal circumference (AC) or estimated fetal weight (EFW) < 10^th^ percentile for gestational age, or by a drop in AC of > 50 percentiles from the measurement at an ultrasound scan performed between 18 and 32 weeks' gestation. The study group consisted of fetuses with late‐onset FGR associated with a genetic syndrome or aneuploidy, structural malformation or congenital infection (anomalous late‐onset FGR) diagnosed antenatally or postnatally. The control group comprised fetuses with non‐anomalous late‐onset FGR. Exclusion criteria were absence of information on perinatal outcome and/or on antenatal or postnatal diagnosis of malformations, aneuploidy, genetic syndrome or infection, thereby preventing classification of the case as anomalous or non‐anomalous late‐onset FGR.

Following the diagnosis of late‐onset FGR, follow‐up scans were scheduled, based on a monitoring protocol shared among the participating units: every 2 weeks in the absence of signs of cerebral blood flow redistribution, defined as cerebroplacental ratio (CPR) > 5^th^ percentile; on a weekly basis in the case of CPR < 5^th^ percentile or mean uterine artery (UtA) pulsatility index (PI) > 95^th^ percentile; and on alternate days in the case of umbilical artery (UA)‐PI > 95^th^ percentile. Delivery was considered from 36 + 0 weeks in the event of UA‐PI > 95^th^ percentile, at 37 weeks in the case of EFW or AC < 3^rd^ percentile, between 37 and 38 weeks in the event of CPR < 5^th^ percentile and from 39 weeks in the absence of any risk factor for adverse perinatal outcome other than suspected late‐onset FGR. The monitoring strategy also included computerized or visual cardiotocography (CTG), which was performed at each assessment. The antenatal ascertainment of any type of anomaly was considered on an individual basis to determine the timing of delivery. The finding of absent or reversed end‐diastolic flow in the UA and spontaneous decelerations of the fetal heart rate represented indications for immediate delivery by Cesarean section. Betamethasone for fetal lung maturity was administered in all cases delivered before 34 + 6 weeks, while pre‐eclampsia represented an indication for delivery at 37 weeks or at 34 weeks in the presence of clinical signs of severity of the disease.

In the event of induction of labor, cervical ripening was promoted by either cervical‐ripening balloon or slow‐release prostaglandin‐E_2_ pessary, followed by administration of oxytocin if onset of labor did not occur. The clinicians responsible for intrapartum care were not blinded to the antepartum findings. A CTG diagnosis of intrapartum fetal distress was defined according to the classification system proposed by the American College of Obstetricians and Gynecologists (ACOG) until 2015 and according to the International Federation of Gynecology and Obstetrics classification after 2015^15^, ^16^. As per standard practice, paired analysis of the cord gases was performed at birth according to the recommendations of the ACOG^17^.

Demographic and clinical data were retrieved from medical records and the ultrasound databases and included maternal age, parity, comorbidities, body mass index at booking and at delivery, diagnosis of hypertensive disorders of pregnancy, gestational age at diagnosis of late‐onset FGR and at delivery, diagnosis‐to‐delivery interval, induction of labor, mode of delivery, fetal biometry (biparietal diameter, head circumference, AC, femur length, and EFW calculated according to the Hadlock IV formula^18^), and fetal (UA and middle cerebral artery) and maternal (UtA) Doppler indices. Neonatal data collected included birth weight, 5‐min Apgar score, UA‐pH, need for admission to the neonatal intensive care unit (NICU), overall length of hospital stay, need for respiratory support or respiratory complications, neonatal jaundice (defined by serum bilirubin > 20.6 mg/dL), neonatal hypoglycemia (defined by two consecutive whole blood glucose measurements by glucometer or one plasma glucose measurement of < 3.3 mmol/L), postnatal survival and other reported perinatal outcomes. Composite adverse perinatal outcome was defined as the presence of at least one of: stillbirth, 5‐min Apgar score < 7, NICU admission, need for respiratory support at birth, neonatal jaundice and neonatal hypoglycemia. The presence of congenital anomalies was ascertained postnatally in neonates with abnormal findings on antenatal investigation or detected after birth. Structural malformations were categorized based on the type of defect into: central nervous system (CNS), cardiac, gastrointestinal, genitourinary, skeletal and thoracic anomalies and multiple anomalies. In cases in which invasive testing for karyotype analysis with or without comparative genomic hybridization (CGH) was not performed prenatally, postnatal testing was at the discretion of the clinician in charge. Congenital infections were diagnosed postnatally by means of polymerase chain reaction performed on different biological samples, irrespective of the results of antenatal investigations if clinically indicated.

The primary aims of the study were to assess the incidence and clinical features of anomalous late‐onset FGR diagnosed between 32 + 0 and 36 + 6 weeks, and to compare the perinatal outcome of such cases with that of fetuses with non‐anomalous late‐onset FGR. Moreover, a subgroup analysis of cases with birth weight < 10^th^ percentile, i.e. small‐for‐gestational‐age (SGA) neonates, was performed.

Statistical analysis was performed using the Statistical Package for Social Sciences (SPSS) version 22 (IBM Inc., Armonk, NY, USA). Categorical data are presented as *n* (%) and comparisons between the anomalous and non‐anomalous late‐onset FGR groups were performed using the χ‐square test. Continuous variables are presented as mean ± SD, with the exception of Apgar scores and length of hospital stay, which are presented as median (range), and comparisons between the two groups were performed using Student's *t*‐test and the Mann–Whitney *U*‐test, respectively; *P* < 0.05 was considered to indicate statistical significance. This study was conducted according to the STROBE guidelines^19^, and it was approved by the local ethics committee of the participating units.

## RESULTS

**Figure 1:**
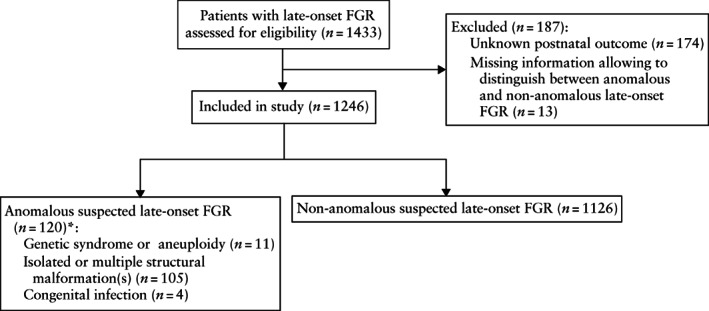
STROBE flowchart showing inclusion of pregnancies between 32 + 0 and 36 + 6 weeks' gestation with suspected late‐onset fetal growth restriction (FGR) with or without associated anomalies. *Associated anomalies (structural defect, genetic abnormality or congenital infection) were confirmed postnatally.

Of 1433 fetuses that fulfilled the study inclusion criteria, 187 were excluded owing to unknown postnatal outcome or missing information related to congenital anomalies, leaving 1246 cases of late‐onset FGR for data analysis. Among these, 120 (9.6%) fetuses were classified as having anomalous late‐onset FGR (Figure 1).

Maternal demographic characteristics and ultrasound findings at diagnosis are summarized in Table 1. The gestational age at diagnosis was lower in fetuses with anomalous than in those with non‐anomalous late‐onset FGR ((33 + 3) ± (1 + 2) weeks *vs* (33 + 6) ± (1 + 3) weeks; *P* = 0.02). The incidence of hypertensive disorders of pregnancy was almost four‐fold higher in fetuses with non‐anomalous late‐onset FGR (9.3% *vs* 2.5%; *P* = 0.01), while there were no differences in the Doppler findings between the two groups.

**Table 1:** Maternal demographic characteristics and ultrasound findings at diagnosis in pregnancies with late‐onset fetal growth restriction (FGR), according to presence of associated anomaly

Characteristic	Anomalous late‐onset FGR (*n* = 120)	Non‐anomalous late‐onset FGR (*n* = 1126)	*P*
Maternal age (years)	31.1 ± 6.0	31.7 ± 5.7	0.23
BMI at booking (kg/m^2^)	21.9 ± 3.7	22.6 ± 4.3	0.10
BMI at delivery (kg/m^2^)	24.2 ± 4.3	25.5 ± 5.0	0.12
GA at diagnosis (weeks)	(33 + 3) ± (1 + 2)	(33 + 6) ± (1 + 3)	0.02
Nulliparous	24/69 (34.8)	264/706 (37.4)	0.69
Hypertensive disorder of pregnancy	3/119 (2.5)	62/670 (9.3)	0.01
EFW (g)	1916 ± 233	1866 ± 285	0.08
EFW percentile	10.3 ± 8.7	11.3 ± 9.7	0.32
Mean UtA‐PI	0.97 ± 0.36	0.89 ± 0.31	0.27
Mean UtA‐PI > 95^th^ percentile	40 (33.3)	304 (27.0)	0.14
UA‐PI	1.05 ± 0.28	1.09 ± 0.27	0.46
UA‐PI > 95^th^ percentile	23 (19.2)	258 (22.9)	0.35
MCA‐PI*	1.76 ± 0.39	1.84 ± 0.44	0.33
Cerebroplacental ratio*	1.66 ± 0.51	1.74 ± 0.57	0.46
Cerebroplacental ratio < 5^th^ percentile*	31/83 (37.3)	346/930 (37.2)	0.98

Data are given as mean ± SD, *n* (%) or *n*/*N* (%).

* Data available for 1013 patients. BMI, body mass index; EFW, estimated fetal weight; GA, gestational age; MCA, middle cerebral artery; PI, pulsatility index; UA, umbilical artery; UtA, uterine artery.

Table 2 summarizes the type and frequency of the anomalies recorded in anomalous late‐onset FGR fetuses, grouped according to genetic syndrome or aneuploidy, structural malformations, and congenital infection. A confirmed genetic or chromosomal condition accounted for 11 (9.2%) cases of anomalous late‐onset FGR. The most common genetic anomaly was trisomy 21 (4/11 cases (36.4%)), followed by sex chromosome defects (2/11 (18.2%)). Structural malformations not associated with genetic syndromes or aneuploidy were recorded in 105 fetuses, accounting for 87.5% of all cases of anomalous late‐onset FGR. The most frequent abnormalities were genitourinary (28/105 (26.7%)), limb (21/105 (20.0%)), cardiac (17/105 (16.2%)), facial (8/105 (7.6%)) and CNS (7/105 (6.7%)) malformations. Congenital infections were recorded in four (3.3%) cases, three of which had cytomegalovirus infection.

**Table 2:** Anomalies diagnosed in 120 fetuses with anomalous late‐onset fetal growth restriction

Type of anomaly	Value
Genetic syndrome or aneuploidy	11 (9.2)
Trisomy 21	4
Klinefelter syndrome	1
Turner syndrome	1
Chromosome 10 deletion	1
Silver–Russel syndrome	1
Aicardi–Goutières syndrome	1
Moebius syndrome	1
Goldenhar syndrome	1
Structural anomaly	105 (87.5)
Central nervous system malformation	7
Ventriculomegaly	4
Microcephaly	1
Partial agenesis of corpus callosum	1
Encephalocele	1
Cardiac malformation	17
Ventricular septal defect	7
Atrial septal defect	6
Tetralogy of Fallot	1
Right ventricular hypertrophy	1
Multiple cardiac malformations	2
Gastrointestinal malformation	8
Gastroschisis	5
Omphalocele	1
Esophageal atresia	1
Intestinal atresia	1
Genitourinary malformation	28
Renal agenesis	6
Hydronephrosis	8
Polycystic kidney	1
Bladder exstrophy	1
Hypospadias	8
Hydrocele	2
Labia majora hypertrophy	2
Facial malformation	8
Cleft lip with or without cleft palate	4
Saddle nose	1
Multiple facial malformations	3
Limb malformation	21
Clubfoot	20
Polydactyly with syndactyly	1
Thoracic malformation	3
Congenital cystic adenomatoid malformation	3
Multiple malformations	11
Other anomaly	2
Thyroid dysgenesis	1
Rectovaginal fistula	1
Congenital infection	4 (3.3)
Cytomegalovirus	3
Toxoplasmosis	1

Data are given as *n* (%) or *n*.

The delivery characteristics and perinatal outcomes of the study groups are shown in Table 3. Fetuses with anomalous late‐onset FGR were more likely to have a birth weight < 3^rd^ percentile (38.3% *vs* 29.2%; *P* = 0.04) and experience composite adverse perinatal outcome (58.3% *vs* 35.9%; *P* < 0.01) than were those with non‐anomalous FGR. Furthermore, fetuses with anomalous, compared with those with non‐anomalous, late‐onset FGR had an increased incidence of need for respiratory support at birth (25.8% *vs* 9.0%; *P* < 0.01), intubation (10.0% *vs* 1.1%; *P* < 0.01), NICU admission (43.3% *vs* 22.6%; *P* < 0.01) and longer hospital stay (24 days (range, 4–250 days) *vs* 11 days (range, 2–59 days); *P* < 0.01). Among fetuses with birth weight < 3^rd^ percentile, those with anomalous late‐onset FGR were more likely to need respiratory support at birth (34.8% *vs* 17.9%; *P* < 0.01) and intubation at birth (10.9% *vs* 2.4%; *P* < 0.01) and had longer hospital stay (27 days (range, 8–250 days) *vs* 15 days (range, 3–59 days); *P* < 0.01) compared with those with non‐anomalous late‐onset FGR (Table S1). One case of stillbirth was recorded in the non‐anomalous late‐onset FGR group.

**Table 3:** Delivery characteristics and perinatal outcome of pregnancies with late‐onset fetal growth restriction (FGR), according to presence of associated anomalies

Characteristic	Anomalous late‐onset FGR (*n* = 120)	Non‐anomalous late‐onset FGR (*n* = 1126)	*P*
GA at delivery (weeks)	(37 + 5) ± (1 + 6)	(37 + 6) ± (1 + 6)	0.24
Induction of labor	48 (40.0)	504 (44.8)	0.32
Male gender	53 (44.2)	591/1110 (53.2)	0.06
Birth weight (g)	2398 ± 493	2429 ± 491	0.34
Birth‐weight percentile	8.8 ± 11.7	9.6 ± 11.2	0.48
Birth weight < 10^th^ percentile	72 (60.0)	751 (66.7)	0.14
Birth weight < 3^rd^ percentile	46 (38.3)	329 (29.2)	0.04
Diagnosis‐to‐delivery interval (weeks)	(4 + 1) ± (2 + 1)	(3 + 6) ± (2 + 2)	0.57
Cesarean section	53/98 (54.1)	379/834 (45.4)	0.11
Delivery before 37 weeks	34 (28.3)	285 (25.3)	0.39
Delivery before 34 weeks	5 (4.2)	49 (4.4)	0.93
5‐min Apgar score*	9 (1–10)	9 (6–10)	0.13
5‐min Apgar score < 7	1 (0.8)	8 (0.7)	0.88
Cord arterial pH†	7.26 ± 0.09	7.28 ± 0.08	0.14
Need for respiratory support at birth	31 (25.8)	101 (9.0)	< 0.01
Neonatal intubation	12 (10.0)	12 (1.1)	< 0.01
Neonatal jaundice	22 (18.3)	144 (12.8)	0.09
Neonatal hypoglycemia	17 (14.2)	164 (14.6)	0.91
NICU admission	52 (43.3)	254 (22.6)	< 0.01
Length of hospital stay (days)	24 (4–250)	11 (2–59)	< 0.01
Composite adverse perinatal outcome‡	70 (58.3)	404 (35.9)	< 0.01

Data are given as mean ± SD, *n* (%), *n*/*N* (%) or median (range).

* Data provided for 1245 cases with live birth. †Data available for 955 cases. ‡Defined as presence of at least one of: stillbirth, 5‐min Apgar score < 7, admission to neonatal intensive care unit (NICU), need for respiratory support at birth, neonatal jaundice or neonatal hypoglycemia. GA, gestational age.

The delivery characteristics and perinatal outcomes of the SGA neonates (birth weight < 10^th^ percentile) are presented in Table 4. SGA neonates with anomalous late‐onset FGR were more likely to experience composite adverse perinatal outcome than were those with non‐anomalous FGR (63.9% *vs* 36.6%; *P* < 0.01). In particular, they had an increased need for respiratory support at birth (27.8% *vs* 9.1%; *P* < 0.01), intubation (11.1% *vs* 1.3%; *P* < 0.01) and NICU admission (47.2% *vs* 20.9%; *P* < 0.01) and longer hospital stay (24 days (range, 4–250 days) *vs* 12 days (range, 2–59 days); *P* < 0.01).

**Table 4:** Delivery characteristics and perinatal outcome of neonates with birth weight < 10^th^ percentile, according to whether they had anomalous or non‐anomalous late‐onset fetal growth restriction (FGR)

Characteristic	Anomalous late‐onset FGR (*n* = 72)	Non‐anomalous late‐onset FGR (*n* = 751)	*P*
GA at delivery (weeks)	(37 + 6) ± (1 + 5)	(37 + 6) ± (1 + 6)	0.98
Induction of labor	29 (40.3)	331 (44.1)	0.54
Male gender	38 (52.8)	351/743 (47.2)	0.37
Birth weight (g)	2405 ± 490	2398 ± 487	0.91
Diagnosis‐to‐delivery interval (weeks)	(4 + 1) ± (2 + 1)	(3 + 6) ± (2 + 2)	0.36
Cesarean section	28/56 (50.0)	267/557 (47.9)	0.77
Delivery before 37 weeks	16 (22.2)	190 (25.3)	0.57
Delivery before 34 weeks	3 (4.2)	33 (4.4)	0.93
5‐min Apgar score*	9 (6–10)	9 (1–10)	0.18
5‐min Apgar score < 7	1 (1.4)	5 (0.7)	0.49
Cord arterial pH†	7.26 ± 0.09	7.28 ± 0.08	0.13
Need for respiratory support at birth	20 (27.8)	68 (9.1)	< 0.01
Neonatal intubation	8 (11.1)	10 (1.3)	< 0.01
Neonatal jaundice	16 (22.2)	109 (14.5)	0.09
Neonatal hypoglycemia	10 (13.9)	112 (14.9)	0.82
NICU admission	34 (47.2)	157 (20.9)	< 0.01
Length of hospital stay (days)	24 (4–250)	12 (2–59)	< 0.01
Composite adverse perinatal outcome‡	46 (63.9)	275 (36.6)	< 0.01

Data are given as mean ± SD, *n* (%), *n*/*N* (%) or median (range).

* Data provided for 822 cases with live birth. †Data available in 651 cases. ‡Defined as presence of at least one of: stillbirth, 5‐min Apgar score < 7, admission to neonatal intensive care unit (NICU), need for respiratory support at birth, neonatal jaundice or neonatal hypoglycemia. GA, gestational age.

## DISCUSSION

### Main findings

The findings of this study demonstrate that anomalous late‐onset FGR accounts for approximately one in 10 cases of FGR diagnosed between 32 + 0 and 36 + 6 weeks' gestation. Most cases of anomalous late‐onset FGR are associated with structural malformations, while genetic syndromes/aneuploidies and congenital infections overall account for just over one in 10 cases. Fetuses with anomalous late‐onset FGR are at increased risk of birth weight < 3^rd^ percentile and composite adverse perinatal outcome at birth, including respiratory complications with need for respiratory support and NICU admission compared with those with non‐anomalous late‐onset FGR. Such findings were confirmed when analyzing the perinatal outcomes of anomalous and non‐anomalous SGA neonates.

### Interpretation of findings in relation to previous knowledge

This research adds to the knowledge about the frequency, type of anomalous findings and perinatal outcome of anomalous late‐onset FGR. Previous studies have shown that anomalous FGR contributes to 15–20% of all cases of FGR^20^, ^21^, ^22^, however, to our knowledge there has been no study describing the actual incidence, clinical features and outcome of FGR first detected between 32 + 0 and 36 + 6 weeks' gestation. Data from a large retrospective cohort of pregnancies with periviable FGR showed that fetal anomalies were present in approximately one in four cases^14^, which is over two‐fold higher than the incidence of anomalous late‐onset FGR. This could be related to the low frequency of genetic conditions and congenital infections reported in fetuses suspected to be small beyond 32 weeks^20^, ^22^.

One of the *a priori* hypotheses of our study was that fetal anomalies are more common and severe when fetal smallness is diagnosed early in gestation^14^, ^23^. In our large cohort of late‐onset FGR fetuses, we found that most anomalies are minor, hence likely to be associated with a favorable prognosis with or without the need for postnatal treatment. With respect to the relationship between structural anomalies and FGR, it has been hypothesized that anomalies in the context of FGR may occur mainly because of environmental factors or misdiagnosed monogenic disorders^24^, ^25^, ^26^.

Our findings showed a higher frequency of hypertensive disorders of pregnancy in non‐anomalous late‐onset FGR, which is not surprising given the fact that placental insufficiency represents the most common etiology of FGR^10^, ^11^. Interestingly, we found no difference in maternal and fetal Doppler studies between anomalous and non‐anomalous late‐onset FGR. This may be explained by the definition of FGR used in our study, which is not consistent with that currently adopted to define FGR secondary to uteroplacental insufficiency^3^, ^27^, hence potentially leading to the inclusion of constitutionally small fetuses in our cohort.

With respect to perinatal outcomes, our data suggest that anomalous late‐onset FGR represents a risk factor for adverse perinatal events leading to NICU admission, particularly related to respiratory morbidity, regardless of the actual size at birth, which is consistent with the findings of previous studies^14^, ^23^. More specifically, the presence of cardiovascular^28^, CNS^29^, gastrointestinal^30^, and renal anomalies^31^, ^32^ has been shown to be associated with an increased incidence of perinatal complications. Such findings remained significant in a subgroup analysis including only SGA neonates.

### Clinical implications

FGR is known to be associated with an increased risk of adverse perinatal outcome^33^, ^34^, ^35^. Data from this study demonstrate that the identification of anomalies in the context of late‐onset FGR is associated with an increased incidence of perinatal complications, a 3‐fold higher incidence of respiratory distress syndrome and a 10‐fold higher frequency of the need for intubation. Such information is important for parental counseling. Furthermore, the antenatal identification and characterization of fetal anomalies is of primary importance for counseling the prospective parents in relation to the long‐term outcome of their child. The latter depends on the underlying etiology and/or type of anomaly associated with fetal smallness. In our cohort, there were no cases of perinatal death in the group with anomalous late‐onset FGR. Furthermore, looking at the etiology of anomalous late‐onset FGR, findings from our study suggest that most of these cases are likely to be associated with a good prognosis in terms of long‐term and intact survival.

The fact that fetal anomalies were found in approximately one in 10 small fetuses supports a systematic assessment of the fetal anatomy in cases of suspected late‐onset FGR. Such an approach is currently endorsed for early‐onset FGR in the guidelines of the International Society of Ultrasound in Obstetrics and Gynecology^27^ and of the Society for Maternal–Fetal Medicine (SMFM)^36^. In this context, recent data have demonstrated the importance of third‐trimester ultrasound in identifying fetal anomalies arising after a normal anomaly scan or overlooked at mid‐trimester fetal anomaly assessment^37^, ^38^. Late‐onset FGR is not regarded as an indication for invasive testing according to the latest SMFM guideline^36^, however, the prenatal identification of fetal anomalies may represent a valid indication to discuss the option of invasive testing for fetal karyotyping with CGH array. Nonetheless, it has to be acknowledged that the failure rate of amniocentesis for fetal karyotyping performed in late gestation is higher than that of the analysis performed at midtrimester^39^, and that the diagnosis of genetic syndromes, aneuploidies or congenital infections by means of invasive testing is worth considering only in settings in which clinical management may differ in the event of abnormal findings.

### Strengths and limitations

To our knowledge, this is the first study reporting on the incidence, etiology and perinatal outcome of late‐onset FGR associated with structural defects, genetic syndromes or aneuploidy or congenital infection. Acknowledging the limitation that not all fetuses and neonates underwent genetic testing, which could have led to the misclassification of some malformations as isolated instead of as part of a genetic or chromosomal anomaly, we believe that our results provide a glimpse of the actual incidence of genetic syndromes or aneuploidies associated with fetal smallness diagnosed between 32 + 0 and 36 + 6 weeks in the context of a developed healthcare system with a systematic policy for the prenatal screening of fetal aneuploidies and structural malformations. The retrospective design of the study and the exclusion of over one in 10 potentially eligible fetuses with suspected late‐onset FGR owing to missing information are among the major limitations of the study. Finally, the criteria adopted to define suspected late‐onset FGR could have led to the inclusion of an undefined number of constitutionally small fetuses, thus underestimating the actual incidence of anomalies associated with late‐onset FGR.

### Conclusions

This study adds to the knowledge about the incidence, clinical features and perinatal outcome of pregnancies with anomalous suspected FGR diagnosed between 32 + 0 and 36 + 6 weeks' gestation. Our findings suggest that fetuses with anomalous late‐onset FGR are more likely to experience respiratory complications after birth and a longer duration of hospital stay following admission to the NICU. Nonetheless, most of the anomalies represent benign isolated structural malformations that are unlikely to impact on the long‐term outcome of the individual. These data could be used for the counseling of prospective parents.

## Supporting information


**Table S1** Delivery characteristics and perinatal outcomes of neonates with birth weight < 3^rd^ percentile according to whether they had anomalous or non‐anomalous late‐onset fetal growth restriction

## Data Availability

Data available on request from the authors.
