# Supplementary material for: Incidence, clinical features and perinatal outcome in anomalous fetuses with late‐onset growth restriction: cohort study
Source: Ultrasound Obstet Gynecol. 2022 Nov 1;60(5):632–9. doi: 10.1002/uog.24961 (PMC9827976; doi:10.1002/uog.24961)
Supplement: Supplementary file 1 — Table S1 Delivery characteristics and perinatal outcomes of neonates with birth weight < 3rd percentile according to whether they had anomalous or non‐anomalous late‐onset fetal growth restriction [file UOG-60-632-s001.docx]

**Table S1** Delivery characteristics and perinatal outcomes of neonates with birth weight < 3^rd^ percentile according to whether they had anomalous or non-anomalous late-onset fetal growth restriction (FGR)

| **Characteristic** | **Anomalous late-onset FGR**  **(n=46)** | **Non-anomalous late-onset FGR**  **(n=329)** | **p** |
| --- | --- | --- | --- |
| GA at delivery, weeks^+days^  (mean ± SD) | 37^+0^± 2^+1^ | 37^+0^ ± 1^+6^ | 0.74 |
| Induction of Labor  N (%) | 11 (23.9%) | 121 (36.8%) | 0.09 |
| Male gender  N (%) | 25 (54.3%) | 161 (48.9%) | 0.50 |
| Birthweight, grams  (mean ± SD) | 1901 ± 402 | 1977 ± 377 | 0.21 |
| Diagnosis-to-delivery, weeks^+days^  (mean ± SD) | 4^+0^ ± 2^+2^ | 3^+2^ ± 2^+0^ | 0.05 |
| Caesarean section  N (%)  *N=262 | 30/39 (76.9%) | 149/223 (66.8%) | 0.21 |
| Delivery <37 weeks  N (%) | 21 (45.7%) | 137 (41.6%) | 0.62 |
| Delivery <34 weeks  N (%) | 5 (10.9%) | 20 (6.1%) | 0.23 |
| 5-min Apgar score^#^  Median (range) | 9 (7-10) | 6 (1-10) | 0.35 |
| Cord arterial pH  (mean ± SD) | 7.27 ± 0.08 | 7.28 ± 0.09 | 0.45 |
| Need for respiratory support at birth  N (%) | 16 (34.8%) | 59 (17.9%) | <0.01 |
| Neonatal intubation  N (%) | 5 (10.9%) | 8 (2.4%) | <0.01 |
| Neonatal jaundice  N (%) | 9 (19.6%) | 69 (21.0%) | 0.83 |
| Neonatal hypoglycemia  N (%) | 10 (21.7%) | 88 (26.7%) | 0.47 |
| NICU admission  N (%) | 25 (58.1%) | 128 (44.8%) | 0.10 |
| Length of hospital admission, days  median (range) | 27 (8-250) | 15 (3-59) | <0.01 |
| Composite adverse perinatal outcome  N (%) | 26 (56.5%) | 163 (49.5%) | 0.38 |

(*) N=375 unless otherwise stated.

^#^Only livebirth.

Data are given as mean ± (SD), number (percentage) or median (range). GA, gestational age; NICU, neonatal intensive care unit.
